# Factors that affect levels of alexithymia, empathy and communication skills of nursing students in northern Iran

**DOI:** 10.1002/nop2.1652

**Published:** 2023-02-20

**Authors:** Rahim Sharafkhani, Ruth Nimota Nukpezah, Remya Lathabhavan, Hakan Kallmen, Andrew Fournier, Zohreh Hosseini Marznaki

**Affiliations:** ^1^ Department of Public health Khoy University of Medical Sciences Khoy Iran; ^2^ Department of General Nursing, School of Nursing and Midwifery University for Development Studies Tamale Ghana; ^3^ Indian Institute of Management Bodh Gaya Bihar India; ^4^ Department of Clinical Neuroscience, STAD, Centre for Psychiatry Research Karolinska Institutet & Stockholm Health Care Services Stockholm Sweden; ^5^ National Park Service Tucson Arizona USA; ^6^ Imam Ali Hospital at Amol City Mazandaran University of Medical Sciences Sari Iran

**Keywords:** alexithymia, communication skills, empathy, mental health, nursing students

## Abstract

**Aim:**

The inability of nurses to express their own emotions, understand others' emotions and show empathy could result in communication gaps that could affect patient care outcomes. This study investigates the factors associated with the levels of alexithymia, empathy and communication skills among nursing students.

**Design:**

A survey was conducted among 365 nursing students, and data were collected using an online questionnaire.

**Methods:**

Data analyses were done using SPSS software version 22.

**Results:**

There was a significant positive association between age and empathy and a negative association between the number of times a nurse took the entrance exam. The level of education and interest in nursing correlate with communication skills. All the predictor variables of alexithymia in this current study were not significant. Emphasis should be placed on improving nursing students' empathy and communication skills. Student nurses should be taught how to recognize and express their emotions. To assess their mental health, they must be screened regularly.

## 1 INTRODUCTION

Nursing students must learn skills such as establishing effective communication, expressing their emotions or thoughts and distinguishing their physical senses from their emotions (Culha & Acaroglu, 2019). Alexithymia hinders people's capacity for empathy and makes encountering communication difficulties inevitable. According to previous investigations, alexithymia has been found to occur at a rate of 10% in healthy persons (Sancar & Aktas, 2019).

Moreover, the demands of the nursing profession, such as workload, and interaction with patients and the environment, add difficulties and stress, which must be tackled with emotional intelligence acumen (Dugué et al., 2021). To facilitate compassionate decision‐making and give effective emotional care, factors like alexithymia, empathy and communication are important and considered crucial in the nursing profession (Giulia et al., 2019).

An individual's ability to understand, express and be aware of emotions is crucial for effective communication (Sancar & Aktas, 2019). Empathy is knowing and understanding other people's feelings and putting oneself in another person's situation. Empathy and emotional awareness are critical in nursing because they are the foundation for understanding patients' needs, concerns and feelings (Hall et al., 2021).

The inability to identify and express emotions is alexithymia (Preece et al., 2020). Alexithymic people cannot perceive and describe their thoughts and feelings and show defects in emotional awareness, insights into their emotions and relationships with others (Alzahrani et al., 2020; Di Lorenzo Rosaria et al., 2019).

While alexithymia is the inability to empathize and relate to others, “emotion without speech” or “lack of words for emotions” indicates difficulty in expressing verbal emotions (Asl et al., 2020), and both are serious issues in the nursing profession. With the shift of medical education from purely theoretical teachings to human communication (Kurtz et al., 2017), alexithymia has become one of the variables used in assessing the competence and ability of nursing students (Expósito et al., 2018).

Many researchers believe that psychological, social and biological factors play a role in alexithymia (Obeid et al., 2020). Recent studies have focused on alexithymia levels or its effects on other variables (Chu et al., 2022; Giulia et al., 2019). Empathy is understanding other people's feelings and putting oneself in another person's situation (Bas‐Sarmiento et al., 2019). Empathy is critical in nursing because it is the foundation for understanding patients' needs, concerns and feelings (Hall et al., 2021). Empathy is essential in the nursing course and professional practice (Bas‐Sarmiento et al., 2019). Since empathy with the patient can lead to better patient satisfaction and treatment (Williams et al., 2016), higher levels of empathy in healthcare staff are needed as it has been associated with improved patient care, greater patient satisfaction and shorter duration of diseases (Giulia et al., 2019; Haley et al., 2017). Studies also revealed that formal training could enhance empathy (Ozcan et al., 2012).

However, the development of empathy skills should not be confined to the education period of master's level nursing students. Still, it must be a continuous process that lasts throughout their professional life (Levett‐Jones et al., 2019). Although empathy has been considered a potential variable over the years in nursing, few studies have discussed the antecedents of empathy (Bas‐Sarmiento et al., 2019; Wu, 2021). An individual's ability to understand, express and be aware of emotions is crucial for effective communication (Sancar & Aktas, 2019). Communication skills are essential for nursing profession because effective communication increases patient satisfaction (Avia et al., 2021), improves treatment outcomes (Molina‐Mula & Gallo‐Estrada, 2020), increases adherence to treatment among patients (Tavakoly Sany et al., 2020) and enhances the quality of care (Molina‐Mula & Gallo‐Estrada, 2020). In contrast, a lack of communication skills can lead to poorer patient compliance with treatment (Lotfi et al., 2019; Molina‐Mula & Gallo‐Estrada, 2020) and inadequate information exchange between patients and health service providers (Lotfi et al., 2019). In establishing effective communication, individuals need to identify and express their feelings to others and be aware that any shortcomings in performing these functions by medical staff could deter communication and negatively affect the quality of patient care (VMK et al., 2019).

Nursing students face challenges in communicating effectively with patients due to various reasons (Arnold & Boggs, 2019). However, the factors or such barriers were not main concerns on the recent studies in this area, rather it was considered as an antecedent for outcomes (Vermeir et al., 2017). The present study considers Stuart stress adaptation model as a grounded theory to explain the theoretical underpinning Stuart stress adaptation Model is a model of psychiatric nursing care, which integrates biological, psychological, sociocultural, environmental and legal‐ethical aspects of patient care into a unified framework for practice (Stuart, 2005, 2014). Connecting with theoretical background, the present study tries to understand the factors of alexithymia, empathy and communication among nursing students considering the biological, psychological, sociocultural and environmental aspects, and consider these assumptions in the behavioural aspects.

With this literature support and to unravel gaps in the present literature, the present study aims to investigate the following:

1. The levels of alexithymia, empathy and communication among nursing students in northern Iran.
2. The factors associated with alexithymia, empathy and communication among nursing students in northern Iran.

Some nursing students face challenges communicating effectively with patients (Arnold & Boggs, 2019). One of the barriers to proper communication between nursing students and patients is their alexithymia, or inability to be aware of their feelings. Alexithymia can also lead to ineffective communication between the nursing staff and patients. Communication skills are essential for nurses (Sancar & Aktas, 2019). Effective communication increases patient satisfaction (Avia et al., 2021), improves treatment outcomes (Molina‐Mula & Gallo‐Estrada, 2020), increases adherence to treatment among patients (Tavakoly Sany et al., 2020) and enhances the quality of care (Molina‐Mula & Gallo‐Estrada, 2020). In contrast, a lack of communication skills can lead to poorer patient compliance with treatment (Lotfi et al., 2019; Molina‐Mula & Gallo‐Estrada, 2020) and inadequate information exchange between patients and health service providers (Lotfi et al., 2019). In establishing effective communication, individuals should identify and express their feelings to others and be aware that any shortcomings in performing these functions by medical staff could deter communication and negatively affect the quality of patient care.

Studies suggest that students with alexithymia cannot empathize with others (Hall et al., 2021). However, empathy plays an essential role in the nursing course and professional practice (Bas‐Sarmiento et al., 2017).

Higher levels of empathy toward patients are required in health care staff because empathy with the patient can lead to better patient satisfaction and treatment (Williams et al., 2016).

Other studies have linked empathy towards patients among health staff to better patient care, higher patient satisfaction and shorter disease duration (Di Lorenzo Rosaria et al., 2019; Haley et al., 2017).

Learning how to empathize is a skill that may be developed through study and practice (Ozcan et al., 2012). However, a meta‐analysis at the University of Michigan that studied the pattern of empathy for the past 30 years showed that today's college students have less empathy than earlier generations. Thus, the development of empathy skills should not be confined to the education period of master's level nursing students but must be a continuous process that lasts throughout their professional life (Levett‐Jones et al., 2019).

Despite the importance of empathy and communication in nursing, few studies have been conducted in this area. The present study investigates the factors associated with nursing students' levels of alexithymia, empathy and communication skills in northern Iran.

## 2 METHODS

### 2.1 Study design

The present study is cross‐sectional and has been conducted to evaluate the factors associated with nursing students' levels of alexithymia, empathy and communication skills in northern Iran.

### 2.2 Samples and research environment

Mazandaran University of Medical Sciences nursing students were included in this cross‐sectional study. The province of Mazandaran is located in northern Iran. This study's research population consists of all students enrolled in the nursing and midwifery programs at Mazandaran University of Medical Sciences. Initial steps first included compiling a complete list of nursing student participants. After compiling a comprehensive list of all nursing and midwifery students enrolled at Mazandaran University of Medical Sciences, we established this cohort as our study population. At first, the investigator interviewed all Educational assistants at Mazandaran's nursing schools. Participants were given an explanation of the study's goals, and their consent was obtained after their understanding of those goals was established. The nursing students' mobile phone numbers were recorded verbally, and a written consent SMS was sent to each of them. After that, an SMS with a link to the online survey was sent to all nursing students. The nursing students were sampled by sending them a message with a link to an online questionnaire via a messaging program. Data were collected from May to October 2021 using the census method. Informed consent was obtained prior to participation. Those who did not give informed consent could not participate in this study. The response rate of the participants was 87%.

### 2.3 Research instruments

The research data were collected using a four‐section instrument, including personal and social information questionnaires – the demographic questionnaire, the Toronto Alexithymia Scale (TAS), the Communication Skills Scale (CSS), and the Scale of Empathy‐Health provider student version (SE‐HPS).

#### 2.3.1 The demographic questionnaire

This section included: age, gender, marital status and level of education. Place of residence, father's job, mother's job, semester, number of times to participate in the entrance exam, work experience in a clinical setting, interest in the nursing profession, smoking and alcohol consumption.

#### 2.3.2 Toronto Alexithymia Scale

This scale was developed in 1985 by Taylor et al. (1985). TAS is a 20‐item test scored on a 5‐point Likert scale. It includes three subscales – difficulty in identifying emotions (seven items), difficulty in describing feelings (five things) and objective thinking (eight items) in a 5‐point Likert scale ranging from completely disagree (score 1) to strongly agree (score 5). A total score is generated for complete alexithymia by adding the scores from the three subscales. The lowest possible score is 20, and the highest is 100. High scores indicate a high level of alexithymia, which indicates severe difficulty in expressing and recognizing emotions. An alexithymia score of 20–40 indicates mild symptoms, 40–60 indicates moderate symptoms, and >60 symptoms severe alexithymia (Besharat, 2007; Giulia et al., 2019). The Iranian version of this scale was prepared by Besharat (2007), and is a good scale. The validity and reliability were assessed in this study (Cronbach's alpha coefficient was 0.90).

#### 2.3.3 Communication Skills Scale

In assessing communication skills, a questionnaire designed by Khaghanizadeh et al. in Iran in 2014 (Khaghanizade et al., 2014) was used. The questionnaire contained 28 items under five areas, vis. “onset awareness” (three items), “verbal and non‐verbal communication skills” (nine items), “external and internal coordination” (six items), “respect for a client” (five items) and “unconditional admission of a client” (five items). On a 5‐point Likert scale, each item was rated as (always, usually, occasionally, rarely and never), ranging from 5 to 1. The minimum and maximum total scores were 28 and 140 respectively. It should be noted that some items of instruments were designed negatively, so they were scored in reverse (items 24, 27 and 28). The student's communication skills level was classified based on the score obtained. Accordingly, students who scored <50% of the total score were classified as weak, students who scored between 50% and 75% were classified as moderate, and students who scored above 75% were classified as good. Internal consistency had Cronbach's alpha value of 0.85, and the split‐half method was reported as (*r* = 0.73; Khaghanizade et al., 2014). The validity and reliability were assessed in this study (Cronbach's alpha coefficient was 0.70).

#### 2.3.4 Scale of Empathy: Health provider student version

The SE‐HPS questionnaire (Fields et al., 2011) was used to assess empathy. This questionnaire included 20 questions about students' empathy when providing health services to patients. These ranged from one to seven based on the degree of agreement; a score of one indicated disagreement (Strongly disagree), and a score of seven indicated complete agreement (Strongly agree). In addition, the questionnaire contained 10 negative items that were scored in reverse order. As a result, each item was scored on a 7‐point Likert scale, and individuals' total scores ranged from 20 to 140. A higher questionnaire score indicated that the individual had a higher level of empathy with the patient and a greater tendency towards empathy in the care setting.

This measure comprised three sub‐scales: perspective‐taking, compassionate care, and patient empathizing scale. Zarifnejad et al. in Iran previously used this questionnaire (Karimi et al., 2018). In a recent review, Hojat and Gonnella (2015) in Iran highlighted the following cut‐offs according to gender: for men, high scores were those that were higher than or equal to 127, and low scores were less than or equal to 95, and for women, high scores ≥129 and low scores ≤100. The total score ranged between 20 and 140, with higher scores pointing to higher levels of empathy (Hojat & Gonnella, 2015). The validity and reliability were assessed in this study (Cronbach's alpha coefficient was 0.87).

### 2.4 Ethical considerations

The Ethics Committee of Mazandaran University of Medical Sciences, Mazandaran, Iran approved this study (IR.MAZUMS.REC.1399.462). All questionnaires were anonymous. The study's aims were explained to the participants who volunteered to participate. For participation, after the participants filled out the informed consent form, the researcher sent the link to the online questionnaire via SMS.

[Correction added on 9 March 2023 after first online publication: The Ethics code was updated as "REC.1399.462".]

### 2.5 Statistical analysis

Descriptive statistics were applied to analyse continuous variables (mean and standard deviations) and categorical variables (% and Chi‐square). Cramer's V criterion was used to investigate the relationship between nominal and ranked qualitative variables. The gamma correlation coefficient was used to examine the correlation between rank qualitative variables. Statistical tests, binary logistic regression and ordinal logistic regression were used to determine the predictor variables. Kolmogorov–Smirnov test showed that the data did not follow the normal distribution; therefore, a non‐parametric test was used.

## 3 RESULTS

The study included 365 students. Approximately 94% of the respondents were between the ages of 19 and 26. The respondents' age means and standard deviations were 22.32 and ± 4.45 years, respectively, and about 50% were men. Of the respondents, 10% percent of the people were married, and the rest were single. More students in the 2nd, 4th and 6th semesters participated in this study. Nearly half (48.8%) of the students had participated twice in the entrance exam. Information about other variables is listed in Table 1.

**TABLE 1:** Demographic characteristics of nursing students (*n* = 365).

	Nursing students (*n* = 365)
Age (year)	22.32 (SD = 4.45)
Sex	
Male	182 (49.86)
Female	183 (50.14)
Marital status	
Single	327 (89.59)
Married	38 (10.41)
Level of education	
BSN	359 (98.36)
MSN	6 (1.64)
Place of residence	
With family	30 (8.22)
Dorm	298 (81.64)
Alone	37 (10.14)
Smoking	
Yes	34 (9.32)
No	331 (90.68)
Alcohol consumption	
Yes	25 (6.85)
No	340 (93.15)
Father's job (*n* = 362)	
Government job	113 (31.22)
Retired	71 (19.61)
Freelance	178 (49.17)
Mother's job (*n* = 362)	
Government job	72 (19.89)
Retired	17 (4.70)
Freelance	273 (75.41)
Semester	4.58 (SD = 2.28)
Number of times to participate in the entrance exam	1.94 (SD = 0.91)
Child rank (*n* = 358)	
1	166 (46.37)
2	110 (30.73)
3	55 (15.36)
4	16 (4.47)
5	7 (1.96)
6	1 (0.28)
7	3 (0.83)
Work experience in clinical setting	
Yes	60 (16.44)
No	305 (83.56)
Lack of interest in the nursing profession	
1	53 (14.52)
2	50 (13.70)
3	5 (1.37)
4	212 (58.08)
5	45 (12.33)

*Note*: Data are presented as number (percentage) and mean (standard deviation).

Abbreviations: BSN, Bachelor of Science in Nursing; MSN, Master of Science in Nursing; SD, standard deviation.

The mean (SD) scores for communication skills, alexithymia and empathy were 89.44 (13.63), 56.78 (12.02) and 82.83 (9.14) respectively. The results showed that the communication skills of half of the participants were poor, and only a quarter of the participants had good communication skills. In addition, two‐thirds of the participants had moderate alexithymia. More detailed data are presented in Table 2.

**TABLE 2:** Mean, standard deviation, and correlations among study variables (*n* = 365).

	Communication skills	Alexithymia	Empathy
Possible range	1–5	1–5	Continuous
Observed range	1–5	1–5	Continuous
Mean (SD)	89.44 (13.63)	56.78 (12.02)	82.83 (9.14)
Frequency: *N* (%)			
Low	180 (49.3)	15 (4.1)	
Moderate	99 (27.1)	239 (65.5)	
High	86 (23.6)	111 (30.4)	
Correlations among outcome variables			
Communication skills	1		
Alexithymia	0.041	1	
Empathy	0.094	−0.024	1
Correlations among study variables with outcome variables			
Age	0.065	0.007	0.002
Semester	−0.172*	−0.018	−0.026
Number of times to participate in the entrance exam	−0.005	0.046	−0.159*

Abbreviation: SD, standard deviation.

* *p* < 0.05.

The hypothesis of normality was rejected via Kolmogorov–Smirnov for all response variables, and therefore, the Spearman correlation coefficient was used. There was a significant inverse correlation between the level of the semester and the level of communication skills, the number of times they wrote the entrance exam, and their levels of empathy (Table 2).

### 3.1 Nominal qualitative variables/dependent variables

There was no statistically significant correlation between nominal qualitative independent variables and rank qualitative dependent variables (Table 3).

**TABLE 3:** Correlations among study variables with communication skills and alexithymia in nursing students.

Variables	Communication skills	Alexithymia
Nominal qualitative variables/dependent variables (rank qualitative); Cramer's value (sig)		
Smoking status	0.50 (0.632)	0.08 (0.349)
Marital status	0.12 (0.090)	0.07 (0.462)
Sex	0.10 (0.141)	0.05 (0.650)
Father's job	0.09 (0.193)	0.06 (0.595)
Mother's job	0.09 (0.171)	0.08 (0.359)
Place of residence	0.06 (0.639)	0.09 (0.198)
Work experience in clinical setting	0.08 (0.317)	0.05 (0.617)
Alcohol consumption	0.06 (0.504)	0.06 (0.494)
Rank qualitative variables/dependent variables (rank qualitative); Gama's value (sig)		
Level of education	0.80 (0.020)*	0.16 (0.701)
Lack of interest in the nursing profession	−0.20 (0.005)*	0.05 (0.566)
Child rank	0.06 (0.374)	−0.05 (0.561)

* *p* < 0.05.

### 3.2 Rank qualitative variables/dependent variables (rank qualitative)

There was a strong correlation between participants' education and communication skills. A statistical test showed that communication skills increased as the education level increased. Interestingly, an inverse relationship was observed between academic satisfaction and communication skills (Table 3).

The relationship between predictor variables and empathy was investigated using binary logistic regression. The empathy variable was divided into two groups. People with empathy scores lower than the median were classified as having low empathy, while the rest were classified as having high empathy. 51% of students were classified as having high empathy. The goodness of fit model was evaluated using omnibus coefficient tests. The chi‐square statistic value for the model with four degrees of freedom was 19,494, which was significant with a *p*‐value of 0.001, indicating that the predictor variables were appropriate. The Hosmer–Lemeshow statistic (8.566 and seven degrees of freedom) demonstrated the model's accuracy in predicting predictor variables (*p*‐value = 0.285). Table 4 demonstrates a significant positive relationship between age and empathy and a negative relationship between age and the number of times the entrance exam was taken. There was, however, no significant relationship between empathy and type or location of residence. With each year of age, the chances of being in the high‐empathy group increased by 11%. However, for each additional entrance exam attempt, the chances of being in the group with high empathy decreased by approximately 77%.

**TABLE 4:** Predictor variables of empathy in nursing students.

Variable	*B*	Std. error	Wald	df	Sig.	OR	95% CI for OR	
							Lower	Upper
Age	0.104	0.044	5.679	1	0.017*	1.110	1.019	1.210
Number of times to participate in the entrance exam	−0.573	0.153	13.964	1	0.001*	0.564	0.417	0.761
Place of residence (with family)	−0.856	0.512	2.798	1	0.094	0.425	0.156	1.158
Place of residence (dorm)	−0.024	0.357	0.004	1	0.947	0.976	0.485	1.966
Constant	−1.094	0.900	1.477	1	0.224	0.335		

*Note*: Place of residence, alone considered as baseline; Statistical test, binary logistic regression.

* *p* < 0.05.

### 3.3 Ordinal logistic regression model

The results showed that when other variables were kept constant, the chance that a participant would have good communication skills (instead of poor or moderate) with increasing semester was about 0.90, which was statistically insignificant. However, by one‐semester increment, the chances of having exceptional or moderate communication skills (instead of poor) tripled, which was statistically significant. An ordinal logistic regression model was fitted to assess predictor variables of alexithymia. According to the findings, none of the predictor variables (marital status, type of residence and the number of times they took the entrance exam) was significant (Table 5).

**TABLE 5:** Predictor variables of communication skill and alexithymia in nursing students.

Variable	Estimate	Std. error	Wald	df	Sig.	OR	95% CI for OR	
							Lower	Upper
Communication skill								
Communication skill (high)	−0.228	0.378	0.365	1	0.546	0.796	0.379	1.668
Communication skill (moderate)	1.004	0.381	6.933	1	0.008*	2.729	1.293	5.760
Semester	0.129	0.045	8.026	1	0.005*	1.137	1.04	1.243
Marital status (married)	−0.331	0.327	1.027	1	0.311	0.718	0.378	1.362
Place of residence (with family)	0.108	0.461	0.055	1	0.815	1.114	0.451	2.745
Place of residence (dorm)	0.490	0.328	2.236	1	0.135	1.632	0.858	3.101
Alexithymia								
Alexithymia (high)	−0.688	0.416	2.737	1	0.098	0.502	0.222	1.135
Alexithymia (moderate)	3.320	0.483	47.230	1	0.001*	27.66	10.729	71.307
Semester	−0.012	0.049	0.059	1	0.808	0.988	0.896	1.088
Marital status (married)	0.198	0.371	0.286	1	0.593	1.218	0.589	2.522
Place of residence (with family)	−0.478	0.496	0.930	1	0.335	0.620	0.234	1.638
Place of residence (dorm)	0.273	0.360	0.574	1	0.449	1.313	0.648	2.661

* *p* < 0.05.

*Note*: Marital status, single considered as baseline; Place of residence, alone considered as baseline; Statistical test, ordinal logistic regression.

## 4 DISCUSSION

This study evaluated the factors associated with nursing students' levels of alexithymia, empathy and communication skills in northern Iran. Nearly half of this study's nursing students had poor communication skills. In contrast to the current study's findings, Ryan et al. showed that the communication skills of medical and nursing students are at moderate to excellent levels. The study further showed that most nursing students had poor communication skills, of which 88.1% needed communication skills training (Xie et al., 2013). Since communication with the patient is one of the critical duties of nurses and leads to the promotion of care, patient recovery, trust, professional development and personality of nurses, paying attention to it, training, maintaining and promoting it in nursing students is very necessary.

More than two‐thirds of the nursing students were seen to suffer from alexithymia. The study by Alzahrani et al. (2020) also showed that half of the medical students suffer from this problem. A study conducted in recent years in Iran among a group of nurses also showed that half of the nurses suffered from this problem (Saeidi et al., 2020). The high rate of alexithymia among these participants could be due to several factors that can increase the risk of mental disorders (Dubey & Pandey, 2013; Sancar & Aktas, 2019).

Anecdotal evidence has shown that alexithymia can affect a person's ability to understand, express and perceive emotions. Alexithymia could affect the critical process of quality and effective communication (Sancar & Aktas, 2019; Tolmunen et al., 2011). Nurses suffering from alexithymia cannot identify their emotional problems, let alone the patient's problems, so early detection and treatment in students can lead to greater patient satisfaction with nursing care (Di Lorenzo Rosaria et al., 2019).

The present study results showed that half of the students had high empathy, and nearly half had low empathy. Results of the study by McKenna et al. (2012) reported the average empathy score in nursing students as being 107.34 ± 13.74. Williams et al. (2014) also reported an average empathy score in nursing students of 104.67. According to these findings, different results are obtained according to the type of questionnaire, society and culture under study.

In agreement with the findings of this current study, many researchers highlight that empathy is an essential ingredient in promoting relationships (Davis, 1990; Dincer & Inangil, 2022; Inslegers et al., 2012; Sevindi & Kumcagiz, 2017), create a climate of trust, facilitate positive patient outcomes, reduce physiological discomfort, improve self‐awareness, etc. According to previous research, alexithymia affects 10% of healthy people (Sancar & Aktas, 2019). Alexithymia can impair a person's ability to empathize, resulting in unavoidable communication difficulties.

The current study did not show a significant correlation between nursing students' empathy, alexithymia and communication skills. In that way, the present study results did not match the results of previous studies. For example, a survey by Sancar and Aktas (2019) showed that as alexithymia increased, communication skills in nursing students decreased. The research results by Karimi et al. (2018) showed that empathy plays an essential role in improving communication skills with the patient and increasing the quality of care, so education in nursing students is necessary. In a study by Graugaard et al. (2004), patients with alexithymia felt more satisfied when receiving a better empathetic response from physicians.

A related study conducted by Pazar et al. (2017) showed that communication skills, psychosocial communication skills and nursing approach courses may not always be influential variables in improving the empathic tendencies of students. The results of the study by Di Lorenzo Rosaria et al. (2019) showed that there is a negative relationship between empathy and alexithymia. The results of studies conducted by Inslegers et al. (2012) showed a negative close relationship between alexithymia and social skills. Hence, people with alexithymia have several problems with communication and interpersonal social skills.

Finally, the results of a study conducted by Sondi and Comkagiz (Sevindi & Kumcagiz, 2017) showed that levels of high alexithymia play a significant role in communication skills. With increasing alexithymia, communication skills decreased, and their research reported an inverse relationship between these two variables. In examining the impact of emotional learning on alexithymia and empathy in nursing students, Dincer et al. found in their clinical investigation that affective learning did influence alexithymia. However, no significant difference was observed between the intervention and control groups regarding empathy (Dincer & Inangil, 2022).

According to most studies, communication skills, psychosocial communication skills and nursing approach courses may not always be influential variables in improving the empathic tendency of students (Pazar et al., 2017). Empathy is unique and different from all other interactions because of two of its characteristics. The first is that we detect empathy after it is realized. The second is that empathy develops in three phases: active listening and self‐transposal, identification and crossing over of self, and sympathy and getting self‐back. People raise their awareness of empathy after it first develops, similar to the feeling of romantic love. There may be no specific sign of empathy before its development, but it is internally recognized after sharing it. Davis has, therefore, indicated that empathy is a more complex process than the individual thinking and feeling as though they have taken someone else's place. Davis states, “If we accept that empathy happens to us, we must accept that it cannot be taught” (Davis, 1990; Pazar et al., 2017). Empathy appears to be a communication process that develops after we are mature enough to receive it. Cognition and emotions develop together during adolescence, and they may have attained the skill to experience empathy. It is possible to help develop empathy, but the behaviour cannot be taught as a skill (Hoffman, 2001; Stietz et al., 2019).

The present study results showed that with increasing levels of education, communication skills also improve. The study conducted by Pazar et al. presented a significant relationship between the semester level and the level of communication skills in students. The level of communication skills increased as the semester progressed (Pazar et al., 2017). There was also an inverse relationship between a lack of interest in nursing and communication skills. The study by Sancar and Aktas (2019) showed a statistically significant difference between selecting a job based on interest or lack of interest and level of communication skills. People who choose their careers with interest showed better communication skills. Admi et al. mentioned that most nursing students who participated in the survey chose their profession enthusiastically. Interest moderates the relationship between alexithymia and communication abilities, even in cases of high alexithymia. The majority of the participants in the study had chosen their field of study with enthusiasm and possessed superior communication skills (Admi et al., 2018).

In this present study, there was a direct relationship between participants' age and empathy. Increased age was directly related to high empathy. Recent studies have also reported a positive relationship between age and empathy (Di Lorenzo Rosaria et al., 2019). With increasing age and school years, empathy also increased. The study results by Williams et al. (2014) showed similar results. However, in some cases, as the academic year progressed, the students' empathy levels decreased (Ward et al., 2012).

However, contrary to the present study's results, the survey by Pazar et al. (2017) showed that the desire for empathy does not increase as the academic year progresses. One conclusion may be that students' clinical experience can be decisive in high empathy scores in the later semesters (Hajibabaee et al., 2018). According to the results, it appears that nursing schools must give empathy training from the beginning.

These results also show that increasing the number of times the entrance exam was taken decreased the chances of empathy. All students take a comprehensive entrance exam to pass their licensing exams in Iran. Hosseini Marznaki et al. showed that most nursing students participated in the comprehensive entrance exam for several years. Most students did not choose the nursing field based on interest. Other conditions have influenced their choice (Marznaki et al., 2021). However, a study by Admi et al. In Israel shows that most nursing students participating in the survey chose to be nurses based on their professional interests (Admi et al., 2018). It can be said that interest in the nursing field can indirectly lead to increased empathy. Therefore, in nursing, special attention should be paid to empathy.

According to the findings, the choice to be a nurse in Iran is influenced by external factors (Marznaki et al., 2021), not merely an interest in the nursing profession and having personality traits, professional tenants and characteristics. Awareness of their feelings allows health professionals to understand the patient's feelings profoundly and helps them establish a respectful therapeutic relationship. Further research is needed to assess better the psychological dimensions of empathy and good patient–nurse communication relationship to implement appropriate and practical education in Iranian university courses for nurses.

### 4.1 Research limitation

The main limitation of the present study was that sampling was limited to nursing students in Mazandaran. Therefore, it is recommended that further studies be conducted on other groups and on a larger scale to generalize the results to other groups. It is possible that conducting a comparison of nursing students with those of other medical and nonmedical students is required to more accurately evaluate the emotional competencies taught in the various courses, which would also suggest the limits and possible outcomes of the teachings as training offered.

## 5 CONCLUSION

According to the current study's findings, those who cannot readily convey their sentiments to others also have difficulties expressing themselves, which is a crucial factor that plays a part in nursing students' ability to communicate effectively. However, it was also observed that most students are ignorant of their high level of alexithymia, which indicates the necessity of follow‐up and counselling by psychiatrists during the study period, and the necessity of locating the source of the problem.

## AUTHOR CONTRIBUTIONS

All authors listed have contributed sufficiently to the project to be included as authors, and all those qualified to be authors are listed in the author by‐line.

## FUNDING INFORMATION

This research received no specific grant from any funding agency in the public, commercial or not‐for‐profit sectors.

## CONFLICT OF INTEREST STATEMENT

To the best of our knowledge, no conflict of interest, financial or other, exists.

## ETHICAL APPROVAL

The Ethics Committee of Mazandaran University of Medical Sciences, Mazandaran, Iran approved this study (IR.MAZUMS.REC.1398.066). All questionnaires were anonymous. The study's aims were explained to the participants who volunteered to participate. For participation, all individuals signed a written informed consent form.

## Data Availability

The data that support the findings of this study are available from the corresponding author upon reasonable request.
